# The Short-Term Consequences of COVID-19 on Mental Health: State of the Art from Available Studies

**DOI:** 10.3390/ijerph192315860

**Published:** 2022-11-29

**Authors:** Gaia Sampogna, Maurizio Pompili, Andrea Fiorillo

**Affiliations:** 1 Department of Psychiatry, University of Campania “L. Vanvitelli”, 80131 Naples, Italy; 2 Department of Neurosciences, Mental Health, and Sensory Organs, Faculty of Medicine and Psychology, Suicide Prevention Centre, Sant’Andrea Hospital, Sapienza University of Rome, 00189 Rome, Italy

Since the outbreak of the COVID-19 pandemic, the consequences on mental health have been found to be considerable, with potential effects on the general population and in high-risk groups, with a variety of physiopathological mechanisms [1,2,3,4,5]. In particular, the “direct effects” include bereavement, loss of social interactions and relationships due to physical distancing and lockdown policies [6], while the “indirect effects” are due to the social uncertainty and economic recession associated with the international health crisis [7]. Further to this, it has been anticipated that the mental health consequences of the pandemic may be even worse in the near future, and are not expected to reduce in the aftermath of this crisis [8,9,10,11,12,13,14,15].

The COVID-19 pandemic has considerably influenced all domains of people’s lives worldwide, causing a high increase in overall psychological distress and several clinical conditions [16,17,18]. The adoption of containment measures has contributed to the occurrence of several stress-related conditions [19] and the deterioration of pre-existing mental conditions [20,21]. The onset of the pandemic was especially stressful for some high-risk groups, such as people with previous severe mental disorders [22,23], migrants [24,25,26], people belonging to ethnic [27,28] or sexual minorities [29,30,31], healthcare professionals, adolescents [32,33,34,35] and pregnant women [36,37,38].

In particular, during the lockdown, higher rates of sleep disorders [39], anxiety, depressive and stress symptoms [40] and higher access rate to mental health services [41] were observed in patients with pre-existing mental disorders. Although containment measures have represented an essential public health strategy to limit the spread of the COVID-19 pandemic, they have significantly affected the mental health of the general population and of people with pre-existing mental health problems. As an integral part of COVID-19 intervention, it would be useful to raise public awareness about the risks of the pandemic with regard to mental health in order to adopt appropriate preventive and therapeutic interventions.

Migrants and those belonging to ethnic or sexual minority groups have been exposed to additional risks to their mental health during the pandemic, due to the combination of the so-called “minority stress” with the additional stress due to the pandemic. In particular, people belonging to minority groups may have experienced difficulties in seeking emotional and practical support from peers due to restrictions on public gatherings, as well as potentially being less well informed on the evolution of the pandemic due to the difficulties in understanding the pandemic-related information released by the media. As pointed out by Yeung et al. [42], it is necessary to understand the specific consequences of the pandemic on the mental health of these high-risk groups as well as to identify specific protective factors [43].

The COVID-19 pandemic is adding further damage to the mental health of healthcare professionals, who especially at the onset of the health crisis were overwhelmed by the exponential increase in workload, with a lack of personal equipment and fear of infection [44,45,46]. All of these elements have contributed to the increasing prevalence of mental health problems and mental disorders in this special target population [47].

Furthermore, an increasing trend in problematic Internet use and excessive video gaming over time in adolescent and young people has been observed [48,49,50]. In a study on the Italian general population, COVID-19-related general psychopathology, stress, anxiety, depression and social isolation played a significant role in the emergence of problematic Internet use, social media addiction and problematic video gaming [51,52,53]. In particular, professional gamers and younger subjects emerged as sub-populations particularly at risk of developing digital addictions. 

As regards pregnant women, the unknown effects of the SARS-CoV-2 virus on pregnancy and the sudden modifications to prenatal care adopted in many countries to reduce viral transmission [37,54,55] represent additional stressful factors to be carefully taken into consideration. The extent to which pandemic-related stress and pregnancy-specific stress have affected mental health in childbearing women during the COVID-19 pandemic is largely unknown. The study by Roslan et al. [56] found that the prenatal ultrasonographic detection of fetal structural anomalies may adversely affect maternal mental health throughout pregnancy, particularly in the current COVID-19 pandemic, in particular within one to two weeks post-detection.

In this Special Issue entitled “The Short-Term Consequences of COVID-19 on Mental Health: State of the Art from Available Studies”, several research papers evaluate and discuss the consequences of the pandemic on mental health from different viewpoints, with contributions from research groups based in different parts of the world, from Hong-Kong to Italy. The contributions vary from the evaluation of psychological factors—such as defense mechanisms, personality traits and mentalization [57]—to the validation of new assessment tools (such as the Adaptation to Change Questionnaire (ADAPTA-10) [58] or the Korean Version of the COVID-19 Phobia Scale (K-C19PS) [59], to the phenomenology of COVID-19 experience in patients with schizophrenia [60].

All of these papers (about 130 at the time when we are submitting this Editorial for the Special Issue) further confirm that the COVID-19 pandemic has represented an unprecedent traumatic event, for which health care professionals were not adequately equipped to manage but have been able to promptly reorganize health care services and the types of interventions offered in order to contain its detrimental impact on mental health [61]. Recognizing and understanding the different pathways underlying the detrimental impact of the pandemic is essential for developing tailored mental health interventions and promoting best practices in healthcare services [62,63,64].

Of course, the pathophysiological mechanisms of the COVID-19 pandemic on mental health may well imply other routes than the psychosocial stressors, including the brain localization of the virus and the effect on the immunoendocrine system [65,66,67]. These aspects are now being investigated as part of Long-Covid Syndrome [68,69,70,71], and will represent the focus of one forthcoming Editorial.
